# Shrinking lung syndrome treated with rituximab in pediatric systemic lupus erythematosus: a case report and review of the literature

**DOI:** 10.1186/s12969-020-00491-0

**Published:** 2021-01-06

**Authors:** Chelsea DeCoste, Dimas Mateos-Corral, Bianca Lang

**Affiliations:** Department of Pediatrics, IWK Health Centre and Dalhousie University, 5850/5980 University Avenue, PO Box 9700, Halifax, Nova Scotia B3K 6R8 Canada

**Keywords:** Rituximab, SLE, Lupus, Child, Lung

## Abstract

**Background:**

Shrinking lung syndrome (SLS), a rare complication of systemic lupus erythematosus (SLE) characterized by dyspnea, low lung volumes, and a restrictive pattern on pulmonary function tests (PFTs), has only been reported in a few children. Given the rarity of SLS there is a paucity of literature regarding its optimal treatment. Outcomes are variable, with case reports documenting some improvement in most patients treated with corticosteroids, with or without additional immunosuppressive agents. However, most reported patients did not recover normal lung function. We report full recovery of a child with SLE and SLS following treatment with rituximab and review the current literature.

**Case presentation:**

An 11-year-old boy presented with a malar rash, myositis, arthritis, oral ulcers, leukopenia, anemia, positive lupus autoantibodies and Class II nephritis. He was diagnosed with SLE and treated with corticosteroids, hydroxychloroquine, azathioprine, and subsequently mycophenolate with symptom resolution. At age 14, his SLE flared coincident with a viral chest infection. He presented with a malar rash, polyarthritis, increased proteinuria and pleuritis which all improved with corticosteroids and ongoing treatment with mycophenolate. Six weeks later he presented with severe dyspnea, markedly decreased lung volumes, but otherwise normal chest X-ray (CXR) and high-resolution chest computed tomography (HRCT). He was found to have severely restricted PFTs (FEV1 27%, FVC 29%; TLC 43%). After additional investigations including echocardiography, pulmonary CT angiography, and diaphragmatic fluoroscopy, he was diagnosed with SLS and treated with rituximab and methylprednisolone. At 1 month his symptoms had improved, but he still had dyspnea with exertion and severely restricted PFTs. At 6 months his FVC and TLC had improved to 51 and 57% respectively, and were 83 and 94% respectively at 4 years. He had returned to all baseline activities, including competitive hockey.

**Conclusions:**

Although extremely rare, it is important to recognize SLS as a possible cause of dyspnea and chest pain in a child with SLE. Optimal treatment strategies are unknown. This is the second reported case of a child treated with rituximab for SLS who recovered normal lung function. International lupus registries should carefully document the occurrence, treatment and outcome of patients with SLS to help determine the optimal treatment for this rare complication.

## Background

Shrinking lung syndrome (SLS) is a rare complication of systemic lupus erythematosus (SLE) characterized by decreased lung volumes and a restrictive pattern on pulmonary function testing (PFTs). Patients typically present with progressive dyspnea and chest pain [1–3]. The diagnosis may be delayed, particularly in pediatric patients, because of failure to consider or recognize this disorder. The exact pathophysiology is unknown [1–3].

Given the rarity of SLS there is a paucity of literature regarding optimal treatment. Case reports and series have documented improvement in most patients treated with corticosteroids, with or without immunosuppressive agents. However, the majority of patients have an incomplete recovery [4, 5]. Recently, rituximab, a monoclonal antibody directed against the CD20 antigen found on the surface of B lymphocytes, has been used successfully in several adult patients and one child with SLE complicated by SLS [6, 7]. We report a child with SLE who developed SLS at age 14 and recovered fully following treatment with rituximab. We also review the literature on SLS in pediatric patients to increase awareness of this exceptionally rare complication, as well as review outcome of patients previously reported with SLS who were treated with rituximab.

## Case presentation

At age 11 years, our patient was diagnosed with SLE after presenting with a malar rash, oral ulcers, polyarthritis, myositis, and anemia. His past history was remarkable only for mild asthma; family history was positive for maternal asthma and hypothyroidism. Work-up revealed leukopenia (WBC 2.5 10e9/L), hypocomplementemia (C3 0.23 g/L), positive anti-nuclear antibody, anti-dsDNA (633 IU/ml; normal less than10), anti-SSa/Ro, and anti-Smith antibodies, Class II lupus nephritis and a SLEDAI of 29. A baseline chest radiograph (CXR) was normal. He was treated with prednisone, hydroxychloroquine (HCQ), and azathioprine. A year later he had no symptoms of his SLE but was evaluated for an exercise-associated cough that was felt to be due to his asthma. A CXR was normal, PFTs showed moderate obstruction and symptoms resolved with a bronchodilator. Over the next few months, despite ongoing HCQ, azathioprine and low dose prednisone he had recurrence of malar rash and mild arthritis; a repeat renal biopsy showed Class III b lupus nephritis and azathioprine was replaced by mycophenolate mofetil (MMF) which was increased up to 700 mg/m2/dose BID, and subsequently decreased due to gastrointestinal side effects. This resulted in resolution of all symptoms but some ongoing nephritis (SLEDAI 12).

At age 14 he was admitted to his local hospital with fever, pleuritic chest pain, dyspnea, and cough. Bilateral pleural effusions were seen on CXR and he was given empiric antibiotics for possible pneumonia prior to transfer to our center, where he received a 3-day pulse of intravenous methylprednisolone (IVMP) for a suspected SLE flare (C3 0.70; anti dsDNA 95; SLEDAI 29). His chest symptoms resolved completely and respiratory syncytial virus (RSV) infection was subsequently confirmed by PCR and was thought to have triggered the flare. One month later, despite treatment with low dose prednisone and MMF (535 mg/m2/dose BID, although compliance was questionable), his SLE again flared. He presented with a malar rash, polyarthritis, as well as findings of pleuritis manifesting with chest pain, dyspnea, and a left pleural effusion. He was treated with pulse IVMP followed by increased MMF (625 mg/m2/dose BID) and daily oral prednisone (1 mg/kg/d).

**Fig. 1:**
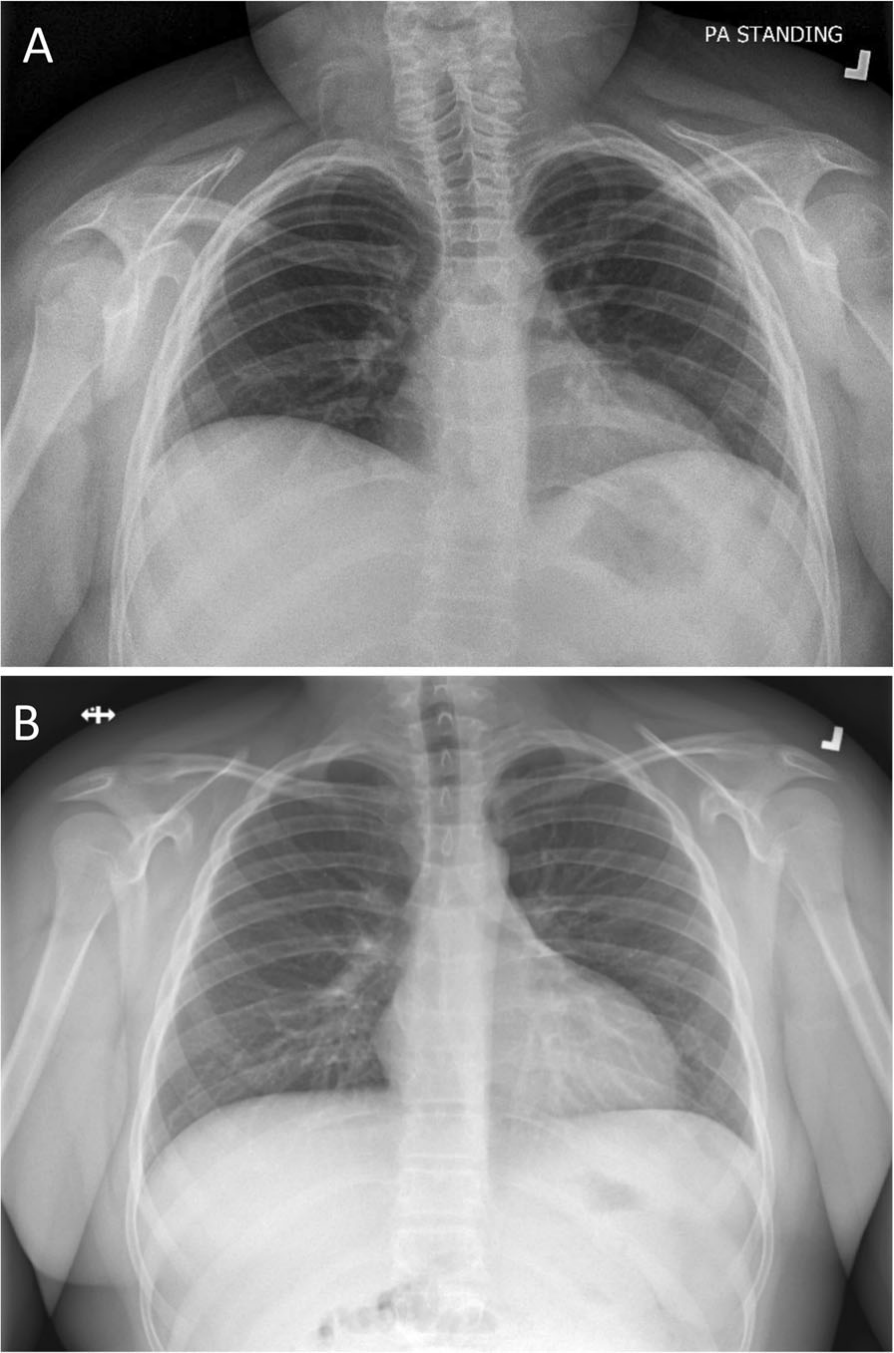
**a** Chest X-ray at presentation with SLS, showing decreased lung volumes and raised hemidiaphragms **b** Normal chest X-ray 3 years later

**Fig. 2:**
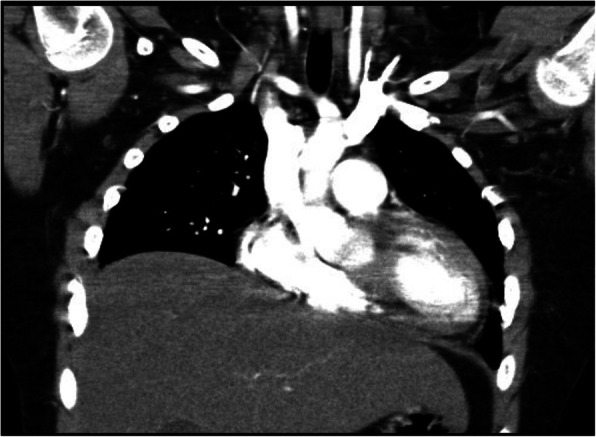
High-resolution computed tomography (HRCT) of the chest at presentation with SLS, showing decreased lung volumes and slight atelectasis

**Fig. 3:**
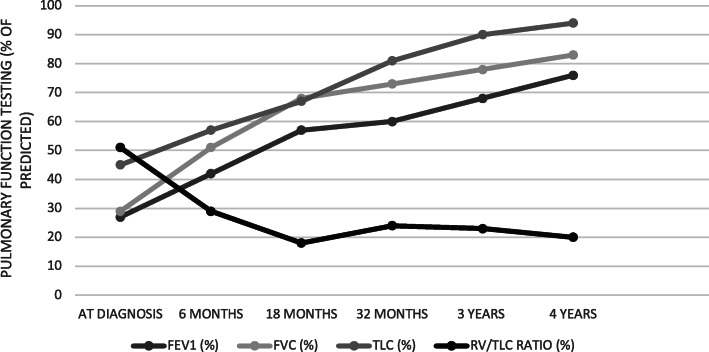
Pulmonary function tests (PFTs) over time in our patient with SLE and SLS; at diagnosis to 4 years following treatment with rituximab. FEV1, forced expiratory volume in 1 s; FVC, forced vital capacity; TLC, total lung capacity; RV, residual volume

Six weeks later his rash and arthritis had improved, but he reported increasing shortness of breath with marked exercise intolerance. On examination he was afebrile, his heart rate was 120, respiratory rate 40, and oxygen saturation 95% in room air. He appeared dyspneic with difficulty speaking in full sentences. Chest examination revealed shallow breathing and decreased air entry bilaterally. Physical examination was otherwise unremarkable. Work up for infection was negative. His SLEDAI had decreased to 14, C3 had normalized (0.97) and anti dsDNA had decreased to 39. CXR and high-resolution computed tomography (HRCT) showed severely reduced lung volumes with no pleural or interstitial disease; there was slight atelectasis but no other significant abnormalities (Figs. 1, 2). An echocardiogram was normal and a CT angiogram showed no evidence of pulmonary embolus. PFTs revealed a severe restrictive pattern with forced expiratory volume in 1 s (FEV1) of 27%, forced vital capacity (FVC) 29%, and total lung capacity (TLC) 43% (Fig. 3). Fluoroscopy documented significantly reduced diaphragmatic movement. He was diagnosed with SLS, and because of worsening dyspnea despite recently increased immunosuppression, he was treated with IVMP 1 g daily for 3 days followed by rituximab 1 g (2 doses, 2 weeks apart). He was also referred to physiotherapy for pulmonary rehabilitation.

One month later he reported some improvement in physical endurance, though he still had dyspnea with mild exertion and PFTs remained severely restrictive. At 6 months, however, he reported much improved exercise tolerance with ability to play some hockey. His FEV1 and FVC had improved to 42 and 51% respectively and TLC had increased to 57%. A planned second course of rituximab, (1 g, 2 doses, 2 weeks apart) was given at 6 months; preceding B cell counts were 8%. He was tapered off corticosteroids over the next 9 months and continued on HCQ (5 mg/kg/d) and MMF (600 mg/m2/dose BID). Serial CXRs showed gradual lung volume expansion. One year after his presentation he returned to playing competitive hockey, with no limitations and no respiratory symptoms. Four years later, he remained asymptomatic with no limitations in activity, no evidence of restrictive lung disease on PFTs (FVC 83%, TLC 94%), and a normal CXR. He remained on the same doses of HCQ and MMF with no clinical evidence of SLE disease activity, normal complement levels (C3 0.97), anti dsDNA 16, a SLEDAI of 2, and a follow renal biopsy showed no active nephritis.

## Discussion

Shrinking lung syndrome is a rare complication of SLE with an incidence of approximately 1% in adult lupus patients [1, 5]. The incidence in pediatric SLE is unknown, but is likely even less common. As such, the diagnosis of SLS may not be considered or recognized in a child with SLE. Clinical features include progressive dyspnea, exercise intolerance, and pleuritic chest pain, all of which were described by our patient. Physical examination frequently reveals tachypnea with decreased air entry and the use of accessory respiratory muscles but is otherwise unremarkable. Findings on CXR often include significantly reduced lung volumes with elevated hemidiaphragms and may include pleural effusions, basal atelectasis, and pleural thickening. HRCT typically shows no parenchymal lung disease, though it may be more sensitive than CXR in demonstrating pleural effusions and atelectasis [4, 8]. PFTs show a restrictive pattern [4]. Reduced diaphragmatic excursion may be demonstrated on chest fluoroscopy, as was seen in our patient, or may be demonstrated using M-mode ultrasonography [8].

The pathogenesis of shrinking lung syndrome remains incompletely understood. When first described by Hoffbrand and Beck in 1965, the condition was thought to be due to surfactant deficiency causing microatelectasis [9]. Since then a number of mechanisms have been suggested, including phrenic nerve dysfunction, pleural inflammation and fibrosis, and diaphragmatic dysfunction due to myositis or neuropathy [3, 10]. Anti-SSa/Ro antibody positivity has been associated with both SLS and myositis, and has been suggested to support the theory that myositis contributes to diaphragmatic dysfunction in some patients [3, 11]. Of note, our patient was anti-SSa/Ro positive and had myositis as part of his SLE course. The frequent occurrence of pleuritic chest pain in up to 80% of patients with SLS has led to a more recently proposed mechanism of pleuritic pain leading to reflex inhibition of diaphragmatic activation and subsequent dysfunction in at least a subset of patients with SLS [1, 3]. Henderson et al. have proposed that pleural inflammation due to the underlying rheumatic disease may lead to activation of neural reflexes which inhibit deep inspiration and cause chronic lung hypoinflation. This is postulated to gradually reduce lung compliance and results in a positive feedback cycle [12]. Interestingly, our patient had developed pleuritic chest pain 2 months prior to his diagnosis of SLS, and had documented pleuritis associated with an RSV infection, and then associated with a flare of his SLE, which may have initiated the pathogenetic process leading to SLS.

There is no standard treatment for patients with SLS. Corticosteroids are the most frequently reported initial treatment of SLS, and can lead to full recovery in some patients [3, 5, 11, 13]. Immunosuppressive agents including cyclophosphamide, azathioprine, and methotrexate are often given along with corticosteroids or if corticosteroids alone are ineffective. There are also a few reports of theophylline and beta-agonists used effectively in SLS, and others advocate the use of analgesia to combat chest pain and pulmonary rehabilitation to improve lung expansion [1, 3, 5, 12, 13]. Although rare fatal cases have been described, the prognosis of SLS in adults is generally considered to be good, particularly in comparison with the progressive course and significant mortality associated with fibrotic interstitial lung disease in patients with SLE [1]. Some clinical improvement has been reported in most patients with SLS, however, recent literature makes it clear that despite treatment many patients do not achieve full recovery. Langenskiold et al., in a review of 35 cases with documented pre and post treatment PFTs, found that only 20% of patients with SLS regained normal lung function [4]. Duron et al. reported full recovery in only a minority of patients in their review of 155 patients with SLS, with 42.9% of patients showing chronic and persistent hemidiaphragm elevation [1]. A report of 20 cases from a large single center in 2018 showed a similar outcome, with 86.7% of patients showing continued restrictive defects on spirometry despite improved lung volumes [5]. The lack of full recovery in both adult and pediatric patients with SLS has led to 15 reports of rituximab use in adults and 2 reports in children with SLS complicating SLE [1, 3, 4, 6–8, 12, 14–16] (Table 1). Normal PFTs were reported in one child treated with rituximab [7], however the other child did not respond [12]. All 4 adult patients with well-documented post-treatment PFTs had normal or near normal findings [3, 4, 6]. Although the degree of objective improvement was not documented in the remaining 11 adult cases treated with rituximab, all were reported to have improved or stabilized. (Table 1). There has also been a recent report of a 19-year-old male with pediatric-onset SLE who developed SLS refractory to IVMP and cyclophosphamide and had some improvement with belimumab, a monoclonal antibody directed against BLyS receptors on B-cells, though long-term follow-up is still ongoing [11].

**Table 1:** Clinical features, treatment and outcome of patients with SLS associated with SLE who received treatment with rituximab

Reference	Sex	Age at SLE diagnosis	Age at SLS diagnosis	Clinical presentation	Imaging findings at SLS diagnosis	PFTs at SLS diagnosis	Treatment	Outcome
[1]	F	Unknown	61	Chest pain, history of pleurisy, dyspnea	Elevated diaphragms, atelectasis, pleural thickening	TLC 46%, DLCO 25%, KCO 59%	CS + Beta-agonists + RTX (dose unknown) + Physiotherapy	Improvement
[1]	F	Unknown	26	Chest pain, history of pleurisy, dyspnea	Pleural thickening, reticulations	FVC 41%, TLC 68%, DLCO 34%	CS + AZA + MMF + RTX (dose unknown)	Improvement
[3]	F	36	46	Dyspnea on exertion, orthopnea, pleuritic chest pain	Elevated diaphragms, atelectasis, pleural thickening	FVC 77%, TLC 68%	CS + CYC + RTX (375 mg/m2 once weekly × 4 q6mo)	Asymptomatic, normal PFTs
[4]	F	28	28 (6 mo after diagnosis of SLE)	Dyspnea, pleuritic chest pain, dry cough, orthopnea	Elevated diaphragms	FVC 61%, TLC 45%	Beta-agonists + theophylline, RTX (1 g × 2, 2 weeks apart) + CYC	Asymptomatic, normal PFTs
[6]	F	38	38^a^	Tachypnea, dyspnea	Normal HRCT, elevated diaphragms	FVC 64%, FEV1 73%	CS + CYC without improvement; followed by RTX (1 g × 2, 2 weeks apart)	Normal PFTs, normal CXR 6 months after treatment
[7]	F	11	14	Dyspnea on exertion, chest pain	Low lung volumes, small bilateral pleural effusions, small pericardial effusion, mild bibasilar atelectasis	FVC 31%, TLC 32%, DLCO 96%	CYC monthly × 1 year, then RTX (dose unknown)	Clinical improvement, PFTs 2 yrs. post: FVC 82%, TLC 80%
[14]	F	22	27	Pleuritic chest pain, exertional dyspnea	Elevated diaphragms, normal HRCT	FVC 1.45 L (predicted value 4.20), TLC 2.35 (predicted value 5.76), DLCO 16.3 (predicted value 26.5)	CS + RTX (375 mg/m2 × 2 doses 6 weeks apart)	Initial clinical improvement, followed by re-presentation requiring second course of RTX. Improvement reported 2 yrs. later
[15]	F	22	57	Dyspnea, dry cough, pleuritic chest pain	Elevated diaphragms, bibasilar atelectasis	FVC 43%, TLC 56%, DLCO 55%	CS + beta-agonists + AZA + MMF, then 6 mo later RTX (1 g × 2 doses, 2 weeks apart, repeated q6mo)	Clinical improvement. PFTs 5 years post: FVC 76%, TLC 79%, DLCO 53%
[16]	F	Unknown	28	Exercise intolerance, pleuritic chest pain	Unknown	FVC 0.99 L	CS + MMF + RTX (2800 mg)	Unlimited exercise tolerance, FVC 2.23 L
[12]	F	12	14	Dyspnea, pleuritic chest pain, orthopnea	Elevated right hemidiaphragm	FVC 36%, TLC 39%, DLCO 102%	CS + RTX (dose unknown) + CYC	Active disease
[8]	F	36	37	Dyspnea, pleuritic chest pain	CXR: Bilateral diaphragmatic elevation with mild pleural effusion HRCT: Mild pleural effusion	Restrictive pattern	CS + MTX + beta-agonists + RTX (dose unknown)	Restrictive defect improvement
[8]	F	36	39	Dyspnea, pleuritic chest pain, fever	CXR: Unilateral diaphragmatic elevation, left atelectasia HRCT: Basal atelectasis	Restrictive pattern	CS + MMF + beta-agonists + RTX (dose unknown)	Restrictive defect stabilization. Developed ILD 4 yrs. later
[8]	F	27	31	Dyspnea, pleuritic chest pain	CXR: Unilateral diaphragmatic elevation, right atelectasia HRCT: Basal atelectasis, mild pleural effusion	Restrictive pattern	CS + theophylline + beta-agonists + RTX (dose unknown)	Restrictive defect stabilization
[8]	F	23	30	Dyspnea, pleuritic chest pain	CXR: Unilateral diaphragmatic elevation HRCT: Basal atelectasis	Restrictive pattern	CS + MMF + beta-agonists + RTX (dose unknown)	Restrictive defect improvement
[8]	F	34	59	Dyspnea, pleuritic chest pain	CXR: Bilateral diaphragmatic elevation, atelectasia HRCT: Basal atelectasis	Restrictive pattern	CS + MMF + RTX (dose unknown) + IVIG	Restrictive defect stabilization

*CS* corticosteroids, *RTX* rituximab, *CYC* cyclophosphamide, *AZA* azathioprine, *MMF* mycophenolate mofetil, *IVIG* intravenous immunoglobulin, *CXR* chest X-ray, *HRCT* high-resolution computed tomography, *PFTs* pulmonary function tests, *FEV1* forced expiratory volume in 1 s, *FVC* forced vital capacity, *TLC* total lung capacity, *DLCO* diffusing capacity for carbon monoxide, *ILD* interstitial lung disease. PFT results expressed in % predicted when available

^a^ Diagnosis of SLS made at the time of diagnosis of SLE

Shrinking lung syndrome is extremely rare in pediatric lupus patients (defined as 16 years or less at diagnosis), with only 6 well-documented case reports identified in our literature review from 1984 to 2019 (Table 2). Age at onset of SLS ranged from 12 to 15 years, 5 were female, all presented with dyspnea, and 5 had associated chest pain. Interestingly, 3 of the 6 presented at the time of diagnosis of SLE, a much more frequent occurrence than that reported in adults with SLS. Of the 6 patients, only 2 reported a return to baseline respiratory function, both clinically and documented on PFTs [2, 7]. In addition to these 6 patients, our literature review identified 3 patients with pediatric onset SLE who developed SLS at age 19 or 20 [11, 19, 20]. Some improvement in lung function was documented in 2 of these patients, one treated with IVMP, cyclophosphamide and azathioprine, and 1 treated with belimumab. In addition, 7 SLE patients 16–18 years of age have been reported, most in case series of SLS, however very limited information was given on their disease course [1, 5, 12, 21, 22].

**Table 2:** Clinical features, treatment, and outcome of reported pediatric cases of SLS associated with SLE

Reference	Sex	Age at SLE diagnosis	Age at SLS diagnosis	Clinical presentation	Imaging findings at SLS diagnosis	PFTs at SLS diagnosis	Treatment	Outcome
[2]	F	12	12^a^	Prior diagnosis of mycoplasma pneumonia with recovery. Re-presented 6 months later with dyspnea	CXR: Enlarged cardiac silhouette, low lung volumes, elevated diaphragms HRCT: thoracic lymphadenopathy	FEV1 34%, FVC 27%, TLC 59%	CS + CYC (q4weeks × 6 mo)	Asymptomatic. Normal PFTs after 1 yr (FEV1 99%, FVC 97%, TLC 92%)
[7]	F	11	14	Dyspnea on exertion, chest pain	CXR: Low lung volumes, small pleural effusions, small pericardial effusion, mild bibasilar atelectasis	FVC 31%, TLC 32%, DLCO 96%	CYC monthly × 1 year, then RTX (dose unknown)	Clinical improvement, PFTs 2 yrs. post: FVC 82%, TLC 80%
[10]	F	15	15^a^	Pleuritic chest pain, dry cough, dyspnea on exertion	Small lung fields, elevated bilateral hemidiaphragms, chest CT normal	FEV1 26%, FVC 25%, TLC 31%	Beta-agonist	Clinical improvement. PFTs after 12d showed FEV1 increase of 58%, FVC increase of 50%, TLC increase of 47%
[17]	M	11	14	Fatigue, dyspnea, pleuritic chest pain	Enlarged cardiac silhouette, atelectasis, severely reduced diaphragmatic excursion on fluoroscopy	FEV1 23%, FVC 20%, TLC 34%	CS + AZA	Follow-up 23 days later: FEV1 45%, FVC 45%, TLC 57%
[18]	F	12	12^a^	Pleuritic chest pain, dyspnea, fever, fatigue, anorexia	CXR: Reduced lung volumes, elevated diaphragms, HRCT: pleural thickening Diaphragmatic fluoroscopy: minimal movement	FVC 39%, TLC 60%, DLCO normal	CS + HCQ	4 yrs. post: ongoing activity limitation, PFTs unchanged
[12]	F	12	14	Dyspnea, pleuritic chest pain, orthopnea	Elevated right hemidiaphragm	FVC 36%, TLC 39%, DLCO 102%	CS + RTX (dose unknown) + CYC	Active disease

*CS* corticosteroids, *RTX* rituximab, *CYC* cyclophosphamide, *AZA* azathioprine, *HCQ* hydroxychloroquine, *CXR* chest X-ray, *HRCT* high-resolution computed tomography, *PFTs* pulmonary function tests, *FEV1* forced expiratory volume in 1 s, *FVC* forced vital capacity, *TLC* total lung capacity, *DLCO* diffusing capacity for carbon monoxide. PFT results expressed in % predicted when available

^a^ Diagnosis of SLS made at the time of diagnosis of SLE

Although extremely rare, it is important to recognize SLS as a possible cause of dyspnea and chest pain in a child with SLE, and be aware that in pediatric patients in particular, this complication may occur at the time of initial presentation of SLE. Our patient clearly stated that his goal for treatment was to return to competitive hockey. Recent literature on rituximab use in SLS, a review of pediatric SLS cases, and our case report suggest that additional therapy, including possible use of rituximab, should be considered in patients with SLS who have an incomplete response to initial immunosuppressive therapy. Careful documentation of the occurrence, treatment and outcome of patients with SLS utilizing large registries of adult and pediatric patients with SLE may help determine optimal treatment for this rare complication.

## Data Availability

Data sharing is not applicable to this article as no datasets were generated or analyzed during the current study.

## Abbreviations

SLS: Shrinking lung syndrome
SLE: Systemic lupus erythematosus
PFT: Pulmonary function test
FEV1: Forced expiratory volume in 1 s
FVC: Forced vital capacity
TLC: Total lung capacity
RV: Residual volume
RSV: Respiratory syncytial virus
CXR: Chest x ray
dsDNA: Double stranded DNA
ANA: Antinuclear antibody
IVMP: Methylprednisolone
PCR: Polymerase chain reaction
HRCT: High resolution computed tomography

## References

[1] Duron L, Cohen-Aubart F, Diot E, Borie R, Abad S, Richez C (2016). Shrinking lung syndrome associated with systemic lupus erythematosus: a multicenter collaborative study of 15 new cases and a review of the 155 cases in the literature focusing on treatment response and long-term outcomes. Autoimmun Rev.

[2] Meinicke H, Heinzmann A, Geiger J, Berner R, Hufnagel M (2013). Symptoms of shrinking lung syndrome reveal systemic lupus erythematosus in a 12-year-old girl. Pediatr Pulmonol.

[3] Toya SP, Tzelepis GE (2008). Association of the shrinking lung syndrome in systemic lupus erythematosus with pleurisy: a systematic review. Semin Arthritis Rheum.

[4] Langenskiold E, Bonetti A, Fitting JW, Heinzer R, Dudler J, Spertini F (2012). Shrinking lung syndrome successfully treated with rituximab and cyclophosphamide. Respiration..

[5] Deeb M, Tselios K, Gladman DD, Su J, Urowitz MB (2018). Shrinking lung syndrome in systemic lupus erythematosus: a single-centre experience. Lupus..

[6] Goswami RP, Mondal S, Lahiri D, Basu K, Das S, Ghosh P (2016). Shrinking lung syndrome in systemic lupus erythematosus successfully treated with rituximab. QJM-INT J Med.

[7] Burns NS, Stevens AM, Iyer RS (2014). Shrinking lung syndrome complicating pediatric systemic lupus erythematosus. Pediatr Radiol.

[8] Borrell H, Narvaez J, Alegre JJ, Castellvi I, Mitjavila F, Aparicio M, Armengol E, Molina-Molina M, Nolla JM (2016). Shrinking lung syndrome in systemic lupus erythematosus: a case series and review of the literature. Medicine..

[9] Hoffbrand BI, Beck ER (1965). “Unexplained” dyspnoea and shrinking lungs in systemic lupus erythematosus. Br Med J.

[10] Thompson PJ, Dhillon DP, Ledingham J, Turner-Warwick M (1985). Shrinking lungs, diaphragmatic dysfunction, and systemic lupus erythematosus. Am Rev Respir Dis.

[11] Ciaffi J, Gegenava M, Ninaber M, Huizinga TW. Shrinking lung syndrome: diagnostic and therapeutic challenges in 3 patients with systemic lupus erythematosus. J Clin Rheumatol. 2019. 10.1097/RHU.0000000000001132. 10.1097/RHU.0000000000001132 31483349

[12] Henderson L, Loring S, Gill R, Liao K, Ishizawar R, Kim S, Perimutter-Goldenson R, Rothman D, Son MB, Stoll M, Zemel L, Sandborg C, Dellaripa P, Nigrovic P (2013). Shrinking lung syndrome as a manifestation of pleuritis: a new model based on pulmonary physiologic studies. J Rheumatol.

[13] Hannah JR, D’Cruz DP (2019). Pulmonary complications of systemic lupus erythematosus. Semin Respir Crit Care Med.

[14] Benham H, Garske L, Vecchio P, Eckert BW (2010). Successful treatment of shrinking lung syndrome with rituximab in a patient with systemic lupus erythematosus. J Clin Rheumatol.

[15] Penacoba Toribio P, Córica Albani ME, Mayos Pérez M, Rodríguez de la Serna A (2014). Rituximab in the treatment of shrinking lung syndrome in systemic lupus erythematosus. Reumatol Clin.

[16] Butterly SJ, Pillans P, Horn B, Miles R, Sturtevant J (2010). Off-label use of rituximab in a tertiary Queensland hospital. Intern Med J.

[17] Ferguson PJ, Weinburger M (2006). Shrinking lung syndrome in a 14-year-old boy with systemic lupus erythematosus. Pediatr Pulmonol.

[18] Krych EH, Fischer PR, Wylam ME (2009). Pleural fibrosis mediates shrinking lungs syndrome in children. Pediatr Pulmonol.

[19] Calderaro DC, Ferreira GA (2012). Presentation and prognosis of shrinking lung syndrome in systemic lupus erythematosus: report of four cases. Rheumatol Int.

[20] Karim MY, Miranda LC, Tench CM, Gordon PA, D’cruz DP, Khamashta MA, Hughes GR (2002). Presentation and prognosis of the shrinking lung syndrome in systemic lupus erythematosus. Semin Arthritis Rheum.

[21] LaRoche CM, Mulvey DA, Hawkins PN, Walport MJ, Strickland B, Moxham J, Green M (1989). Diaphragm strength in the shrinking lung syndrome of systemic lupus erythematosus. Q J Med.

[22] Pillai S, Mehta J, Levin T, Muzumdar H, Nandalike K (2014). Shrinking lung syndrome presenting as an initial pulmonary manifestation of SLE. Lupus..

